# Mathematical modeling reveals that metabolic feedback regulation of SnRK1 and hexokinase is sufficient to control sugar homeostasis from energy depletion to full recovery

**DOI:** 10.3389/fpls.2014.00365

**Published:** 2014-07-28

**Authors:** Thomas Nägele, Wolfram Weckwerth

**Affiliations:** Department of Ecogenomics and Systems Biology, University of ViennaVienna, Austria

**Keywords:** plant systems biology, energy metabolism, *Arabidopsis thaliana*, sugar metabolism and signaling, mathematical modeling, hexokinase, SnRK1

## Abstract

Sucrose and trehalose-6-phosphate (T6P) are central compounds in the regulation and orchestration of whole plant metabolism, growth, development, and flowering. To evaluate their highly complex and regulatory interaction with the two conserved sugar and energy sensors Snf1-related protein kinase 1 (SnRK1), an AMPK-related protein kinase, and hexokinase (Hxk), we developed a kinetic model which demonstrates the subtle metabolic control of sugar homeostasis in a wide range of concentrations without the need for changes in gene expression or protein concentrations. Our model approach is based on a comprehensive set of published metabolite concentrations under various conditions and coupled enzyme kinetics accounting for the role of SnRK1 and Hxk in the sugar and energy homeostasis. This allowed us to investigate interactions between sugar phosphates, such as T6P, which are metabolic inhibitors of SnRK1 and Hxk, and sucrose synthesis during the transition from carbon deficiency to availability. Model simulations and sensitivity analyses indicated that slight changes in SnRK1 activity induced by allosteric effectors may be sufficient to explain a dramatic readjustment of metabolic homeostasis. This may comprise up to 10-fold changes in metabolite concentrations. Further, the Hxk/T6P/SnRK1 interaction implemented in the model supports the interpretation of phenotypic and transcriptomic changes observed in Hxk overexpressing plants. Finally, our approach presents a theoretical framework to kinetically link metabolic networks to underlying regulatory instances.

## INTRODUCTION

Plant carbohydrates are the primary products of photosynthesis and are thus central to regulation of metabolism, development and growth. Carbohydrates are synthesized as triose phosphates in chloroplasts. The carboxylation of ribulose-1,5-bisphosphate generates an instable C6 compound that decomposes into two molecules of glycerate-3-phosphate. Under consumption of NADPH and ATP being generated by the photosynthetic electron transport chain, glycerate-3-phosphate is reduced to give triose phosphates, which can either be exported to the cytosol or be metabolized in the chloroplast as a substrate for starch synthesis (Heldt et al., 2011). Cytosolic triose phosphates are substrate for sucrose synthesis for which fructose-1,6-bisphosphatase (cFBPase) and sucrose phosphate synthase (SPS) have been identified as rate limiting (Stitt et al., 1983; Strand et al., 2000). Sucrose may then be transported to sink organs or it is cleaved by invertase to glucose and fructose (Sturm, 1999). Free hexoses are phosphorylated in a reaction catalyzed by hexokinase (Hxk). The hexose phosphates are substrates for sucrose synthesis, glycolysis or the synthesis of organic and amino acids (Talts et al., 2004; Claeyssen and Rivoal, 2007; Ainsworth and Bush, 2011).

Beside their metabolic interaction via enzymatic interconversions, plant sugars act as signaling molecules. Analyses of the *Arabidopsis* glucose insensitive 2 mutant (*gin2*), which lacks the catalytic activity of a specific hexokinase (HXK1), have identified AtHXK1 as a central component in plant sugar sensing and signaling (Moore et al., 2003). Hxk is present in various cellular compartments, also comprising the nucleus (Yanagisawa et al., 2003). HXK1 was suggested to form a glucose signaling complex core together with the vacuolar H^+^-ATPase B1 and the 19S regulatory particle of proteasome subunit, RPT5B (Cho et al., 2006). In context of its metabolic activity, mitochondrial Hxk together with mitochondrial and/or cytosolic invertase activity was discussed to contribute to the homeostasis of reactive oxygen species (ROS; Camacho-Pereira et al., 2009; Xiang et al., 2011). Finally, due to its connection and interaction with hormone signaling pathways (Rolland et al., 2006; Hanson and Smeekens, 2009), it becomes obvious that Hxk plays various complex roles in metabolism of the whole plant.

In addition to Hxk, there are several other central regulatory instances linking sugar metabolism to signaling networks and energy metabolism. One prominent example is the Snf1-Related Protein Kinase-1 (SnRK1; AKIN10/11) which has a catalytic domain similar to that of Snf1 (Sucrose non-fermenting-1) of yeast and AMPK (AMP-activated protein kinase) of animals (Hardie, 2007). It has been shown that SnRK1 is involved in the regulation of a broad array of target genes which orchestrate the response of metabolism to starvation (Baena-Gonzalez et al., 2007). Central plant proteins identified as direct substrates for SnRK1 are 3-hydroxy-2-methylglutaryl-coenzyme A (HMG-CoA) reductase, nitrate reductase (NR), 6-phosphofructo-2-kinase/fructose-2,6-bisphosphatase (F2KP), TPS and SPS [for an overview see (Halford et al., 2003) and (Halford and Hey, 2009)]. SnRK1 phosphorylation results in inactivation of these enzymes, yet inactivation of NR, F2KP and TPS also requires the binding of a 14-3-3 protein (Bachmann et al., 1996; Moorhead et al., 1996; Halford and Hey, 2009).

Energy deprivation induces a comprehensive SnRK1-mediated transcriptional and metabolic reprogramming. Hence, it is not surprising that SnRK1 activity is regulated on various levels of molecular organization. Recently, (Rodrigues et al., 2013) showed that two clade A type 2C protein phosphatases (PP2Cs) dephosphorylated and inactivate SnRK1. As PP2Cs are repressors of the abscisic acid (ABA) pathway this indicates a way how cellular metabolism is connected to plant hormonal interactions affecting the whole plant metabolism, development and growth. An activation of SnRK1 in *Arabidopsis* has been shown to be due to two upstream kinases, SnRK1-activating kinases (SnAK) 1 and 2 (Crozet et al., 2010).

Beyond the regulation of SnRK1 by other kinases and phosphatases, the inhibition of SnRK1 by metabolites, and predominantly by intermediates of the central carbohydrate metabolism, has been shown to have significant impact on its activity. The phosphorylated sugars trehalose-6-phosphate (T6P), glucose-1-phosphate (G1P), glucose-6-phosphate (G6P) as well as ribose-5-phosphate (R5P) and ribulose-5-phosphate (Ru5P) were shown to reduce SnRK1 activity significantly while fructose-6-phosphate (F6P) and uridine-5′-diphosphoglucose (UDPG) did not inhibit SnRK1 (Nunes et al., 2013b). Particularly the T6P/SnRK1 interaction has been focused in various recent studies. T6P was shown to play a crucial role in regulation of carbon utilization and growth in *Arabidopsis thaliana* (Schluepmann et al., 2003). Hence, like SnRK1, it represents a regulatory instance which is likely to exert major control on plant metabolism and development. In plants, T6P is synthesized from UDPG and G6P. This reaction step is catalyzed by trehalose phosphate synthase (TPS) and enzyme TPS1 is discussed to account for most TPS activity in plants (Vandesteene et al., 2010). In a subsequent step, T6P is converted to trehalose (Tre) in a reaction catalyzed by trehalose phosphate phosphatase (TPP). Finally, trehalase catalyzes the cleavage of Tre in two Glc equivalents. Recently, TPS1 was suggested to be required for timely initiation of flowering in *Arabidopsis thaliana* (Wahl et al., 2013). In another study, the reaction product of TPS1, T6P, was shown to respond significantly to external Suc supply when Suc was fed to seedlings (Lunn et al., 2006). Yet, Nunes and co-workers discussed T6P not to be a growth signal *per se*, but rather to prime gene expression through SnRK1 for growth in response to Suc accumulation under sink-limited conditions (Nunes et al., 2013a).

**FIGURE 1:**
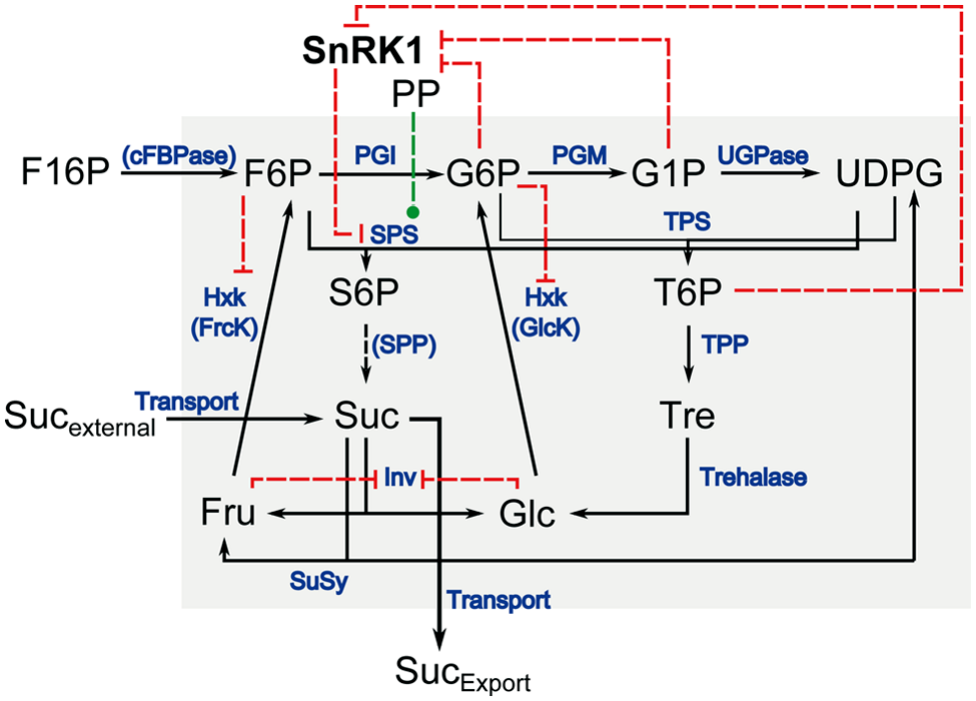
**Schematic overview of the cytosolic carbohydrate metabolism in *Arabidopsis*.** Enzymatic interconversions are indicated in black arrows, enzymes that catalyze each step are written in blue. Red dashed lines indicate inhibitory interaction, green dashed lines indicate activation. SnRK1: Snf1-related protein kinase 1; PP: protein phosphatase; F16P: fructose-1,6-bisphosphate; F6P: fructose-6-phosphate; G6P: glucose-6-phosphate; G1P: glucose-1-phosphate; UDPG: uridine-5′-diphosphoglucose; S6P: sucrose-6-phosphate; T6P: trehalose-6-phosphate; Suc: sucrose; Tre: trehalose; Fru: fructose; Glc: glucose; Suc_external_: externally supplied sucrose; Suc_Export_: sucrose exported to sinks; cFBPase: cytosolic fructose-1,6-bisphosphatase; PGI: phosphoglucoisomerase; PGM: phosphoglucomutase; UGPase: uridine-5′-diphosphoglucose pyrophosphorylase; SPS: sucrose phosphate synthase; TPS: trehalose phosphate synthase; SPP: sucrose phosphate phosphatase; TPP: trehalose phosphate phosphatase; Hxk: hexokinase; GlcK: glucokinase; FrcK: fructokinase; Inv: invertase; SuSy: sucrose synthase.

Assembling this information about the interaction of the central carbohydrate metabolism and its interaction with SnRK1 yields a complex picture of metabolic regulation. Even a simplification of this network which only accounts for the kinase interaction of SnRK1 with SPS, which does not require the binding of a 14-3-3 protein for its inactivation, and including inhibitions exerted by metabolites on SnRK1 but also on Hxk (Claeyssen and Rivoal, 2007) and invertase (Inv; Sturm, 1999), still yields a complex model of the (cytosolic) carbohydrate metabolism (**Figure 1**). Particularly under changing environmental conditions, for example the addition of sucrose to starved seedlings, it is hardly possible to intuitively draw a conclusive and comprehensive picture about regulatory consequences yielding the adjustment of a metabolic homeostasis. Focusing the question how SnRK1 can functionally be integrated in the regulation of the central carbohydrate metabolism in terms of enzyme kinetics, we present an approach of kinetic modeling which allows for the comprehensive analysis of the SnRK1/C-metabolism interaction during a repletion experiment of C-starved seedlings of *Arabidopsis thaliana*. Our approach accounts for the experimental findings of various studies, which have been published previously, and provides a representative and systematic overview of central metabolic consequences emanating from SnRK1 and Hxk activity.

## RESULTS

### A KINETIC EQUATION FOR SnRK1 ACTIVITY

To estimate the impact of SnRK1 on the central carbohydrate metabolism and, *vice versa*, to analyze how its activity is affected by dynamic changes of metabolic inhibitor concentrations, a kinetic equation was derived to simulate the rate *v*_SnRK1_ by which SPS is phosphorylated and inactivated (Eq. 1).

(1) $$v_{SnRK1}=\frac{v_{\max,SnRK1}\,.\,\left[E_{SPS,act}\right]\,.\,\left(1+k_{partial}\,.\,\frac{\left[T6P\right]}{K_{i,T6P}}\right)}{K_{M,E_{SPS,act}}+\left[E_{SPS,act}\right]\,.\,\left(1+\frac{\left[T6P\right]}{K_{i,T6P}}+\frac{\left[G1P\right]}{K_{i,G1P}}+\frac{\left[G6P\right]}{K_{i,G6P}}+\frac{\left[G1P.T6P\right]}{K_{i,T6P}.K_{i,G1P}}+\frac{\left[G6P.T6P\right]}{K_{i,T6P}.K_{i,G6P}}\right)}\left(1\right)$$

**FIGURE 2:**
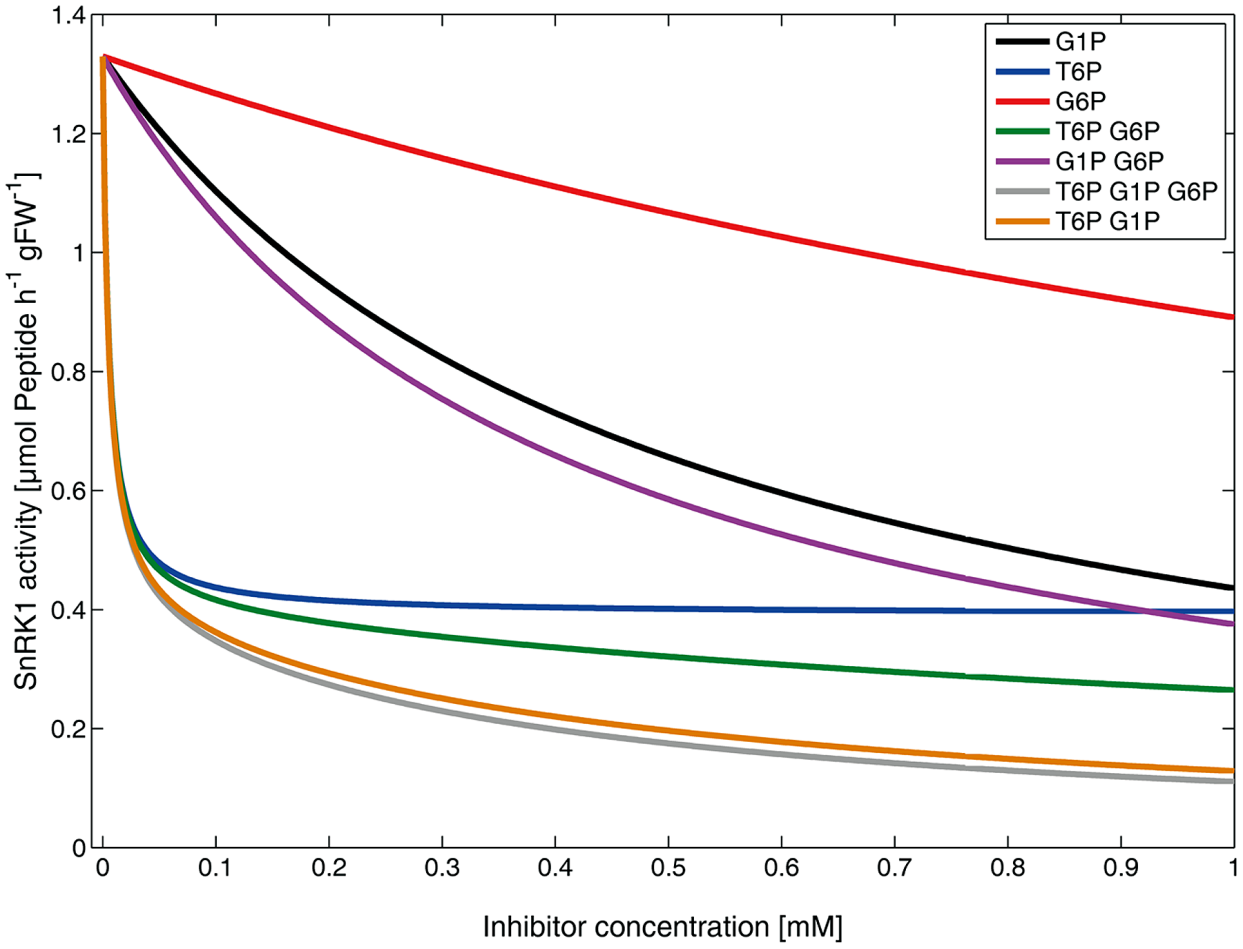
**Simulated kinetic effects of metabolic inhibitors on SnRK1 activity.** Lines represent the rate of phosphorylation by SnRK1 (*v*_SnRK1_), i.e., the solutions of equation (1), under various inhibitor concentrations and combinations.

Kinetic parameters, i.e., *v*_max,SnRK1_, *k*_partial_, *K*_M,ESPS,act_, *K*_i,T6P_, *K*_i,G1P_ and *K*_i,G6P_ were estimated and derived from literature (see Materials and Methods). *E*_SPS,act_ represents the concentration of activated dephosphorylated SPS peptide which was the substrate for SnRK1. As SPS is phosphorylated and inactivated by SnRK1 at Ser-158 (Halford et al., 2003), the phosphorylation stoichiometry was 1. Simulation of *v*_SnRK1_ under single and combined inhibitor concentrations ranging from 0 to 1 mM finally resulted in inhibitor kinetics (**Figure 2**) which were highly similar to the experimental findings reported by Nunes et al. (2013b).

Increasing T6P induced a very fast decline of *v*_SnRK1_ but was asymptotically stable at a value of about 28% of the *v*_max,SnRK1_. Increasing G1P levels resulted in a significant reduction of *v*_SnRK1_ which was, however, not as pronounced as that of T6P. The increase of G6P showed least impact of these three metabolic inhibitors on *v*_SnRK1_ which fully agrees with the experimental findings (Nunes et al., 2013b). Combinations of T6P, G1P, and G6P revealed the highest impact on *v*_SnRK1_ by a T6P–G1P–G6P combination resulting in a final activity of about 10% of the *v*_max,SnRK1_ when all inhibitors had a concentration of 1 mM. This effect was the strongest, followed by T6P–G1P (∼12% of *v*_max,SnRK1_), T6P–G6P (∼18% of *v*_max,SnRK1_) and G1P–G6P (∼28% of *v*_max,SnRK1_). These findings are also highly similar to the previously published experimental results.

### EMBEDDING OF SnRK1 ACTIVITY IN THE CENTRAL CARBOHYDRATE METABOLISM

A mathematical model, based on a system of ODEs, was programmed according to the graphical model structure shown in **Figure 1**. A detailed description of the model and its design is provided in the section “Materials and Methods.” The functional connection of SnRK1 and SPS activity was arranged by two ODEs describing the time-dependent dynamics of concentrations of phosphorylated (inactivated) and dephosphorylated (activated) SPS peptide, i.e., SPS protein with one phosphorylation event per protein. Activated SPS peptides were substrate for SnRK1 and simultaneously defined the maximum enzyme activity of SPS (*v*_max,SPS_) as described in Eq. 2. Model simulations aimed at the reproduction of a short-term sugar feeding experiment with C-starved *Arabidopsis* seedlings (Yadav et al., 2014). Hence, the simulation time equaled the time period of external supply of C-starved seedlings with 15 mM sucrose. The initial conditions were given by the metabolite levels of seedlings before the start of sucrose supply. Except for trehalose, activated and inactivated SPS peptide all initial concentrations were taken from the study of Yadav et al. (2014). The initial trehalose content was estimated to be in the range of nM/low μM (Paul et al., 2008). The inactivated and activated SPS peptide amount was estimated from data reported by Lehmann et al. (2008): from a total amount of SPS protein, *E*_SPS,total_ ≈ 8.5 × 10^-6^ μmol gFW^-1^, we assumed that C-starved seedlings compared to non-C-starved seedlings have a higher activity of SnRK1, and hence the initial condition for activated SPS, *E*_SPS,act_, was determined to be significantly lower than for inactivated SPS, *E*_SPS,inact_. Based on the comparison of SPS activity during day (high energy supply) and night (lower energy supply; Nägele et al., 2010, 2012), initial *E*_SPS,act_ of C-starved seedlings were estimated to be about 15–25% of *E*_SPS,total_ resulting in an amount of *E*_SPS,act_ ranging 1.275 to 2.125 × 10^-6^ μmol gFW^-1^.

### MODEL SIMULATIONS REVEAL A VERY FAST REGULATION OF SPS INDUCED BY THE DYNAMICS OF METABOLIC INHIBITORS OF SnRK1

**FIGURE 3:**
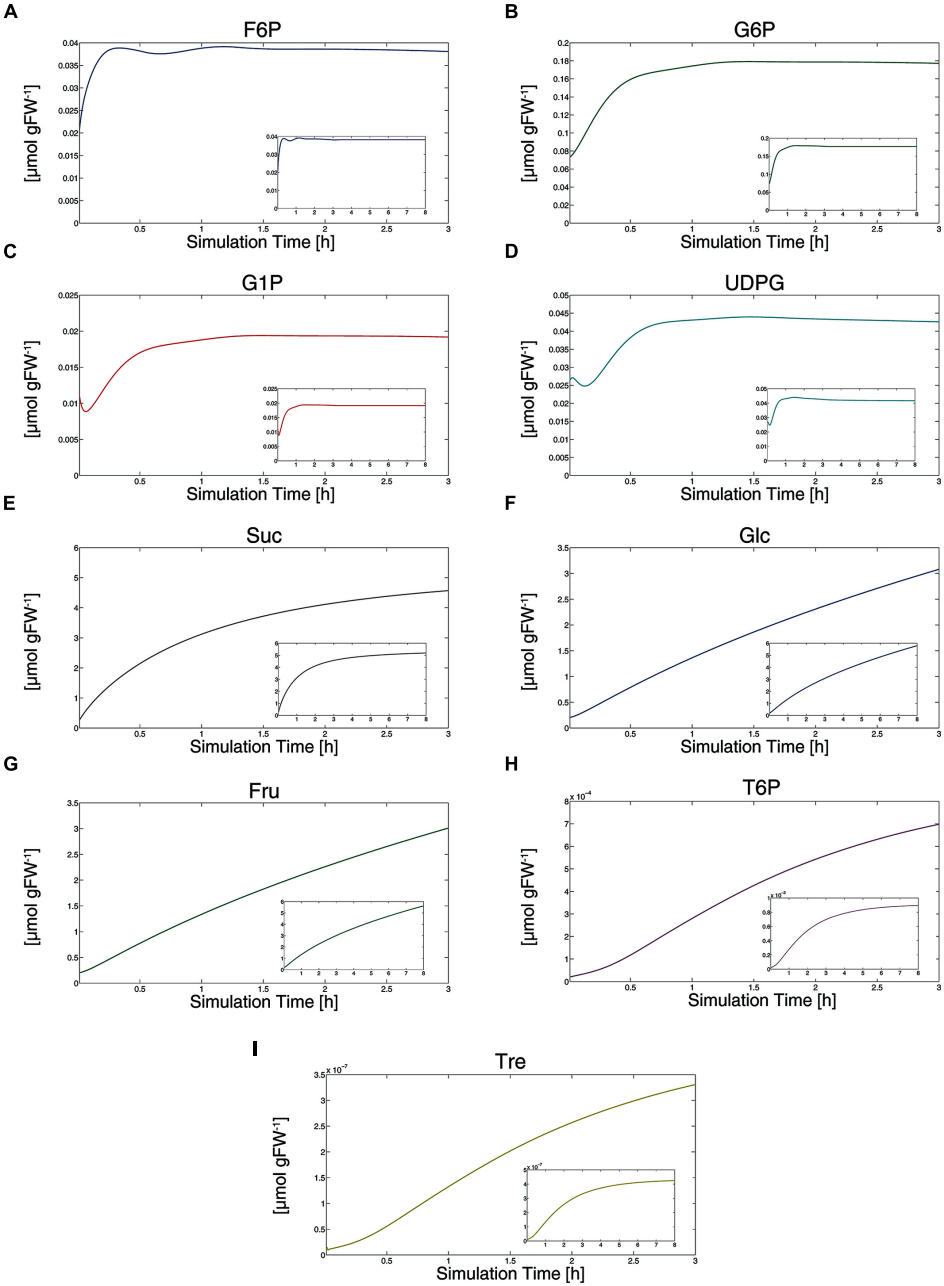
**Simulation of metabolite dynamics induced by external sucrose supply.** Results of simulations are provided for all metabolites **(A–I)** within a simulation period of 3 h (large diagrams) and 8 h (small diagrams). Except for Tre **(I)**, simulations of 3 h directly refer to experimental findings of Yadav et al. (2014).

Simulating metabolite dynamics during an external sucrose supply over 3 h resulted in metabolite dynamics (**Figure 3**) which were highly similar to previously published experimental findings (Yadav et al., 2014). All metabolite levels showed a significant increase during the first 30 min of external sucrose supply. Most of the metabolite pools increased following a saturating course, while particularly free hexoses, i.e., glucose and fructose, increased linearly (**Figures 3F,G**). To test whether our model and its underlying kinetic parameters and assumptions were also valid beyond a simulation time of 3 h, we simulated an external 15 mM sucrose supply for 8 h which has been experimentally performed by Lunn et al. (2006). Although absolute metabolite levels differed between the two studies of Yadav et al. (2014) and Lunn et al. (2006) our model simulations resulted in the same qualitative metabolic changes as described by both of them: only hexose levels show a linear increase even after 8 h of external sucrose supply (**Figure 3**, small diagrams). These simulation results indicate that our proposed model successfully describes metabolite dynamics ranging from C-starved to sugar supplied seedlings.

Simulation results further indicated the level of trehalose to be in a low range compared to other metabolite levels (**Figure 3K**). While we have no explicit experimental evidence for that simulation result, it is in agreement with the finding of Müller et al. (2001) who could not determine trehalose in leaf tissue of *Arabidopsis*, indicating a very low absolute amount compared to other carbohydrates.

**FIGURE 4:**
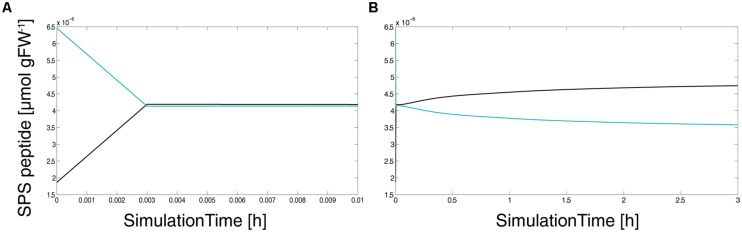
**Simulated time course of activated and inactivated SPS peptide.** Black lines indicate activated/dephosphorylated SPS peptide, turquoise lines indicate inactivated/phosphorylated peptide. **(A)** shows the first 0.01 h after simulated external sucrose supply, **(B)** shows the whole simulation period of 3 h.

A central output of our model simulations was the occurrence of a very fast and linear activation step of SPS which was due to an inactivation of SnRK1 by the increasing levels of its metabolic inhibitors (**Figure 4**). Simulations revealed a time for activation of about 0.003 h or 11 s (**Figure 4A**), i.e., the first molecule of the external supply enters the intracellular reaction network and it takes about 11 s until *E*_SPS,act_ equals *E*_SPS,inact_. The second activation step was slower and showed an asymptotic course (**Figure 4B**) finally resulting in about 60% activated SPS peptide (4.75 × 10^-6^ μmol gFW^-1^ of 8.2 × 10^-6^ μmol gFW^-1^).

### THE INTERACTION OF T6P, SnRK1 AND SUCROSE

**FIGURE 5:**
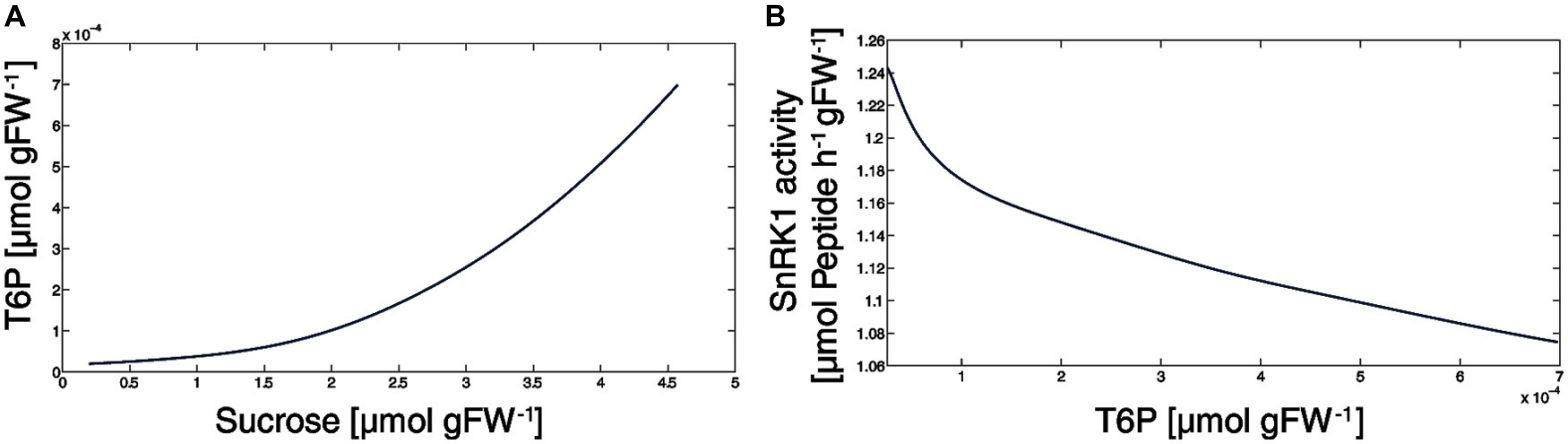
**The SnRK1-T6P-Sucrose interaction. (A)** The T6P level first increases exponentially, then linear with the sucrose level. **(B)** The decrease of SnRK1 activity with respect to T6P concentrations occurring during 3 h of external sucrose supply.

Current models for the role of T6P in plant growth and metabolic regulation discuss T6P to be closely related to sucrose availability and to inhibit SnRK1 which increases the expression of biosynthetic genes, e.g., Nunes et al. (2013a). In this context, we have analyzed the simulated T6P versus sucrose content and found, as already expected from the experimental data (Yadav et al., 2014), a corresponding picture (**Figure 5A**). Particularly in the sucrose concentration range 0.25–3.5 μmol gFW^-1^, the T6P level increased exponentially while it became almost linear at higher sucrose concentrations (3.5–4.6 μmol gFW^-1^). These higher sucrose levels emerged after at least 1.25 h of external sucrose apply (**Figure 3E**). At this time point, also the reaction rate of SnRK1 became linear with respect to T6P concentrations (**Figure 5B**) indicating a tight connection of T6P and sucrose via SPS and SnRK1.

**FIGURE 6:**
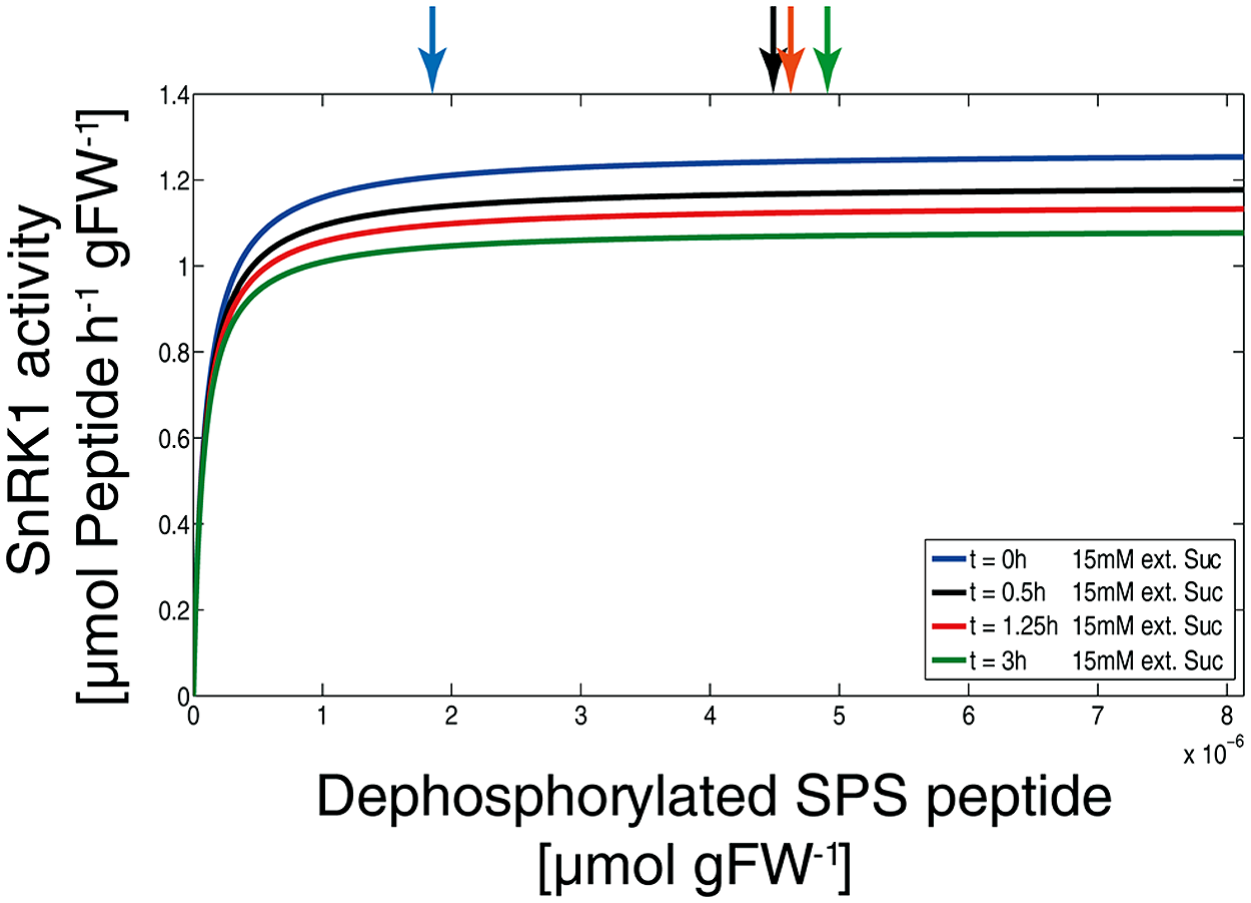
**SnRK1 kinetics under various physiological conditions.** Simulations of SnRK1 kinetics were performed solving equation (1) with the simulated levels of metabolic inhibitors as they are presented in ****Figure 3****. Arrows on the top indicate the level of substrate, i.e., of dephosphorylated SPS peptide, at the time points 0 h (blue), 0.5 h (black), 1.25 h (red), and 3 h (green) after external sucrose supply (15 mM).

To evaluate how the SnRK1 kinetic is affected at different time points of external sucrose supply and under physiological conditions, the simulated concentrations of metabolic inhibitors were determined and *v*_SnRK1_ was calculated (**Figure 6**). Due to the partial and non-competitive inhibition of SnRK1 there is no change in its substrate affinity but only in the *v*_max,SnRK1_. These simulations indicate that a change from *v*_SnRK1_ = 1.2 before sucrose supply to *v*_SnRK1_ = 1.0 μmol Peptide h^-1^ gFW^-1^ after 3 h of sucrose supply can explain the observed metabolic changes by a reduced rate of SPS phosphorylation, i.e., inactivation.

### STEADY-STATE SENSITIVITY ANALYSIS REVEALS A DIFFERENT BEHAVIOR OF GLUCO- AND FRUCTOKINASE DUE TO PERTURBATIONS IN EXOGENOUS SUGAR SUPPLY

Due to the observed linear increase of free hexoses, which was also occurring after 8 h of simulation time, a steady-state of the system of ODEs, i.e., d/d*t* = 0 for all metabolite levels, could not be determined. There are several explanations for such a behavior and one of them is a missing element in the model structure, for example a subcellular compartment for metabolite storage like the vacuole. In a previous study, Kruger et al. (2007) have already demonstrated the impact of the vacuolar glucose pool on the calculation of steady-state fluxes in heterotrophic *Arabidopsis* tissues. In the vacuolar compartment, hexoses cannot be phosphorylated due to a missing hexokinase activity. At the same time, a different invertase isoenzyme with different biochemical properties is active (Sturm, 1999). Hence, we tested whether we could identify a metabolic steady-state after 3 h of external sucrose supply by variation of invertase-related parameters (*v*_max_, K_M_, K_i_). This approach succeeded and a metabolic steady-state could be identified with a reduced *v*_max_ and an increased K_M_ of invertase.

In previous studies, hexokinase (Hxk) has been shown and discussed to be a central component in plant sugar signaling and perception [an overview is provided in (Rolland et al., 2006)]. To analyse a possible interaction between the SnRK1-related and the Hxk-related regulatory influence on the central carbohydrate metabolism, a steady-state sensitivity analysis was performed focusing on changes which are induced by a perturbation of the external sucrose supply. The regulatory impact of Hxk on the metabolic homeostasis was estimated by an *in silico* overexpression of Hxk activity, i.e., the *v*_max_ of glucokinase and fructokinase were increased threefold. In this way, a possible limitation of sugar metabolism by the hexose phosphorylation rate would be significantly decreased and the susceptibility of other subsequent metabolic steps to a change in external sucrose supply could be identified. Ratios of sensitivities calculated for the overexpressed and the wild type model are provided in **Table 1**.

**Table 1:** Changes in sensitivities to perturbation in external sucrose supply due to Hxk overexpression.

Model states/reaction rates	Sensitivity ratio Hxk ox vs WT
*v*_SPS_	7.42
UDPG	5.83
*v*_FrcK_	2.92
T6P	2.14
Tre	2.14
*v*_TPS_	2.1
*v*_TPP_	2.1
*v*_TH_	2.1
F6P	1
Suc	1
Fru	1
*v*_Suc.import_	1
*v*_cFBPase_	1
*v*_PGI_	1
*v*_Suc.Export_	1
*v*_SuSy_	1
*E*_SPS.act_	0.93
*E*_SPS.inact_	0.9
*v*_SnRK1_	0.9
*v*_PP_	0.9
G6P	0.18
G1P	0.18
*v*_PGM_	0.18
*v*_UGPase_	0.18
*v*_GlcK_	0.071
*v*_Inv_	0.07
Glc	0.061

*Ratios are sorted in a descendent order. *v*_x_: rate of the reaction catalyzed by enzyme x; all other metabolite and protein abbreviations refer to the legend in ****Figure 1****.*

The comparison of steady-state sensitivities revealed an articulate increase of sensitivities of *v*_SPS_, UDPG, *v*_FrcK_, as well as all compounds and reactions of the T6P/Tre metabolism due to the increased *v*_max_ of gluco- and fructokinase. The strongest decrease in sensitivities was observed for *v*_GlcK_, v_Inv_ and Glc. At this point, it is interesting to note that although both metabolic hexokinase-associated activities, gluco- and fructokinase, were simulated with a threefold increase in *v*_max_, the sensitivities of both reaction rates behaved differently – sensitivity of *v*_FrcK_ increased while the sensitivity of *v*_GlcK_ decreased.

## DISCUSSION

Sensing and integrating environmental stimuli is a prerequisite for the successful adaptation of organisms to a changing environment. Due to their sessile lifestyle, particularly plants have developed various strategies to cope with environmental dynamics. Central and conserved mechanisms, such as the SNF1/AMPK/SnRK1 protein kinases and the sugar sensor hexokinase, have been shown to play a crucial role in regulation of metabolism, development and stress response (Rolland et al., 2006; Polge and Thomas, 2007). In recent years, numerous comprehensive studies have focused on analyzing the role of SnRK1 in the reprogramming of metabolism. SnRK1 was identified to be significantly involved in linking stress, sugar, and developmental signals, thus playing a pivotal role in the global regulation of plant metabolism, energy balance and growth (Baena-Gonzalez et al., 2007). In addition, central metabolic signaling pathways comprising the sucrose and T6P metabolism have recently been discussed to have a major impact on metabolic and developmental homeostasis (Yadav et al., 2014). Although these – and numerous other – studies provide compelling evidence for a highly efficient, interlinked and central regulatory network, its function *in vivo* and, particularly, its properties under physiological and dynamic conditions is still poorly understood. Beyond, it is still not clearly understood how SnRK1 activity affects the metabolic reprogramming during stress conditions, for example C-starvation. While it is known that low levels of glucose, high levels of sucrose and darkness induce the activity of SnRK1 (Rolland et al., 2006), a comprehensive characterization also accounting for the differential impact of T6P, G1P, and G6P on SnRK1 activity (Nunes et al., 2013b) is difficult to obtain. This is mainly due to the complexity of the underlying metabolic and signaling networks comprising numerous components and a vast amount of regulatory interactions. During the last decade, mathematical kinetic modeling has emerged as an attractive approach to analyse such networks which produce outputs that can hardly be assessed by intuition. Here, we applied such an approach to the sucrose/T6P metabolism by connecting SnRK1 with SPS activity in a mathematical model based on enzyme kinetics. Although our model was solely based on allosteric and posttranslational modifications, and did neither include transcriptional regulation of genes encoding enzymes of carbohydrate metabolism nor activation/inactivation of SnRK1 by SnAKs or PP2Cs, we could successfully simulate the experimental output from previous studies on C-starved *Arabidopsis* seedlings which were fed with sucrose (Lunn et al., 2006; Osuna et al., 2007; Yadav et al., 2014). While this model is far from being complete because it focuses on central interactions of proteins and metabolites and disregards various regulatory instances, it is remarkable that comprehensive and significant metabolic changes can be accounted for. This indirectly supports the suggestion of Nunes et al. (2013a) who discussed T6P not to be a growth signal *per se* but to prime various processes, such as gene expression, through SnRK1. This indicates that, despite the compelling experimental evidence for multiple interlaced regulatory circuits involving the transcriptome, proteome and metabolome, the regulation of a metabolic homeostasis under certain physiological conditions may involve only a subset of the available regulatory instances. Finally, this again emphasizes the necessity of modeling approaches due to the diversity of explanations for metabolic regulation originating from the non-linear relationship between different levels of molecular organization.

Our simulations show that a slight reduction (<20%) of SnRK1 activity, exerted by its metabolic effectors, is sufficient to explain a dramatic reprogramming of the central carbohydrate metabolism (**Figure 6**). Additionally, sensitivity analysis provided evidence that the phosphorylation/dephosphorylation step of SPS was indispensable for a successful simulation (data not shown) because slight modification (1%) of SnRK1- and PP-related parameters had a dramatic effect on the model output. Finally, our simulations revealed that small variations in T6P concentration had a large impact on sucrose content (**Figure 5A**), particularly in the physiological range of 0.5–3 μmol Suc gFW^-1^. This highly sensitive T6P-sucrose interaction sheds light on previous findings which showed T6P to be indispensable for carbohydrate utilization and, as a consequence, growth in *Arabidopsis thaliana* (Schluepmann et al., 2003): sucrose represents the major transport sugar which is, at the same time, central to numerous regulatory processes (Koch, 2004) and stabilizes metabolism during environmental perturbation (Nägele et al., 2010).

Further, it is interesting to note that above a T6P level of 0.3–0.5 nmol gFW^-1^ and a sucrose level above 3.5 μmol gFW^-1^, the relationship of both metabolites was almost linear (**Figure 5A**). In the same concentration range, also the SnRK1 activity behaved linear with respect to T6P (**Figure 5B**), indicating an indirect regulation of sucrose by the T6P-SnRK1 interaction. This might deliver the kinetic explanation for a previous finding, where increases of sucrose above this level were found to result in a proportionate increase both in T6P level and changes in expression of SnRK1 marker genes (Nunes et al., 2013a).

A major difference of allosteric regulation compared to transcriptional regulation is the time frame in which regulation finally takes place. Our calculations revealed that the initiation phase of SPS activation by reduction of its phosphorylation required 10–15 s after external sucrose supply to C-starved seedlings which makes it a quite efficient regulatory system capable of a fast response to fluctuating environmental conditions. Hence, although T6P and the reaction rates involved in its metabolism represent only a fraction of those found in sucrose metabolism (Paul et al., 2008), we provide further evidence for its highly sensitive regulatory impact on sucrose metabolism.

Addressing the question how two conserved central signaling mechanisms – T6P/SnrK1 and Hxk – might be coordinated within the central carbohydrate metabolism, we performed an *in silico* overexpression of Hxk, i.e., GlcK and FrcK activity. Hexokinase activity represents a rate limiting step in sucrose cycling, i.e., the cleavage and re-synthesis of sucrose. Previously we could show that it participates significantly in the stabilization of the central carbohydrate metabolism due to environmental perturbations (Nägele et al., 2010; Henkel et al., 2011). Beyond its catalytic activity, HXK1 was shown to be a glucose sensor integrating nutrient, light, and hormonal signaling (Moore et al., 2003). Hence, *in silico* overexpression of Hxk activities increased the maximum capacity of hexose phosphorylation, thus reducing its rate limitation. The finding that, as a consequence of increased Hxk activities, the whole T6P metabolism was affected more than twice as much as in the wild type model under external perturbations (**Table 1**) indicated a possible interdependency of Hxk- and T6P-dependent signaling pathways. Additionally, the strongest effect of environmental perturbation was observed for sucrose synthesis, catalyzed by SPS, which is directly related to the T6P sensitivities by SnRK1. Yet, as the phosphorylation rate of SnRK1, *v*_SnRK1_, was only slightly affected in its sensitivity, the main perturbation of the T6P/SnRK1/SPS interaction might rather be related to the UDPG pool. Due to being a substrate for both SPS and TPS, changes in the UDPG pool as well as in its sensitivity and stability are also affecting the pathways of sucrose and T6P synthesis.

Based on our findings we hypothesize that an increase of Hxk activity, e.g., by AtHXK1 overexpression, might induce fluctuations in the T6P metabolism which can directly, and very fast, affect SnRK1 activity and/or other signaling compounds which have not been analyzed here. In this context, previous studies reporting on AtHXK1 overexpression in tomato and *Arabidopsis* found a decreased expression of photosynthetic genes and an inhibition of growth (Dai et al., 1999; Kelly et al., 2012). This physiological output is similar to the output being characteristic for SnRK1 activity (Baena-Gonzalez et al., 2007), and hence it supports a model of Hxk/T6P/SnRK1-interdependent signaling.

## MATERIALS AND METHODS

### MODEL DRAFT

A model of the central cytosolic carbohydrate metabolism in plants was conceptualised comprising the enzymatic interconversion of the metabolite pools of fructose 6-phosphate (F6P), glucose 6-phosphate (G6P), glucose 1-phosphate (G1P), uridine 5-diphosphoglucose (UDPG), trehalose 6-phosphate (T6P), trehalose (Tre), sucrose 6-phosphate (S6P), sucrose (Suc), glucose (Glc) and fructose (Fru). One model input was defined by the rate of interconversion from fructose 1,6-bisphophate (F16P) to F6P which is catalyzed by cytosolic fructose 1,6-bisphosphatase (cFBPase). cFBPase activity was shown to be rate limiting for sucrose biosynthesis (Strand et al., 2000), and hence subsequent reactions catalyzed by the enzymes phosphoglucoisomerase (PGI), phosphoglucomutase (PGM) and UDPG pyrophosphorylase (UGPase) were assumed to be not rate limiting (Meng et al., 2009). We are aware that this represents a strong simplification of the *in vivo* system, which, in addition to cFBPase, also comprises the enzymatic activity of inorganic pyrophosphate-dependent diphosphate-fructose-6-phosphate 1-phosphotransferase (PFP) and ATP-dependent phosphofructokinase (PFK). Yet, as our approach focuses on the subsequent steps of the cFBPase/PFP/PFK-driven reaction, we omitted these reaction kinetics and the regulatory impact of the fructose-6-phosphate 2-kinase/fructose-2,6-bisphosphate 2-phosphatase reaction to reduce uncertainties in the biochemical model being caused by structure, parameters and kinetics (Schaber et al., 2009). To simulate the effect of external sucrose on the central carbohydrate metabolism, a second model input was defined by a Michaelis–Menten kinetic equation for sucrose transport which was directly connected to the intracellular Suc pool. The model structure is shown in **Figure 1**. Based on the physical and kinetic evidence for an association of SPS and sucrose phosphate phosphatase (SPP; Echeverria et al., 1997), it was assumed that free S6P was not occurring, and the interconversion of S6P to Suc was simulated in one step with the reaction catalyzed by SPS (indicated by dashed line in **Figure 1**). Rates of metabolite interconversion were modeled by Michaelis-Menten kinetics except for the reactions catalyzed by PGI, PGM, UGPase, TPP and sucrose synthase (SuSy). These reaction rates were assumed to be not limiting and to follow a mass-action kinetic, i.e., to be directly proportional to the substrate concentration. The reaction rate of sucrose cleavage, catalyzed by the invertase (Inv) enzyme, was modeled as an irreversible Michaelis-Menten kinetic incorporating mixed inhibition by the reaction products Glc (non-competitive inhibitor) and Fru (competitive inhibitor; Nägele et al., 2010). Reaction rates of hexose phosphorylation, catalyzed by the enzyme hexokinase (Hxk) with a fructokinase and glucokinase activity, were modeled as Michaelis-Menten enzyme kinetics non-competitively inhibited by F6P (fructokinase) and G6P (glucokinase; Claeyssen and Rivoal, 2007). All other reactions were modeled by irreversible non-inhibited Michaelis-Menten kinetics.

### INTEGRATION OF SnRK1 ACTIVITY IN SPS KINETICS

To kinetically integrate the phosphorylation and inactivation of SPS by SnRK1, the maximum enzyme velocity of SPS, *v*_max,SPS_, was described as the product of the rate constant *k*_SPS_ and the absolute amount of dephosphorylated and, hence, activated SPS peptide, *E*_SPS,act_ (Eq. 2):

(2) $$v_{\max,SPS}=k_{SPS}\,\;.\;\,E_{SPS,act}$$

The rate constant k_SPS_ and the total enzyme level of SPS *E*_SPS,total_, i.e., the sum of *E*_SPS,act_ and *E*_SPS,inact_, were estimated from previous publications (Osuna et al., 2007; Lehmann et al., 2008). We estimated *v*_max,SPS_ = 422 nmol gFW^-1^ min^-1^ = 25.32 μmol gFW^-1^ h^-1^ (Osuna et al., 2007), and *E*_SPS,total_ = 8.5 pmol gFW^-1^ = 8.5 × 10^-6^ μmol gFW^-1^ (Lehmann et al., 2008). Based on these data we estimated the rate constant k_SPS_ (Eq. 3):

(3) $$k_{SPS}=\frac{v_{\max,SPS}}{E_{SPS,total}}=2.98\times 10^{6}.\frac{1}{h}$$

Then, *v*_max,SPS_ was integrated in a bi-substrate kinetic to describe the reaction rate of SPS (Eq. 4):

(4) $$v_{SPS}=v_{\max,SPS}\,\cdot \,\frac{\left[F6P\right]}{K_{M,F6P}+\left[F6P\right]}\,\cdot \,\frac{\left[UDPG\right]}{K_{M,UDPG}+\left[UDPG\right]}$$

Dynamics in levels of activated enzyme *E*_SPS,act_ and of inactivated enzyme *E*_SPS,inact_ were described by ordinary differential equations (ODEs) depending on the phosphorylation by SnRK1 and the dephosphorylation by a protein phosphatase PP (Eq. 5):

(5) $$\begin{array}{c} d[E_{SPS,act}]/dt=v_{PP}-v_{SnRK}\\ d[E_{SPS,inact}]/dt=v_{SnRK}-v_{PP} \end{array}$$

The rate of SPS activation by dephosphorylation was modeled via an irreversible Michaelis-Menten kinetic in which *E*_SPS,inact_ was the substrate. The rate of SPS inactivation by phosphorylation was modeled by a SnRK1 kinetic equation in which *E*_SPS,act_ was the substrate. The derivation of this kinetic equation is described in the following section.

### DERIVING A KINETIC EQUATION FOR SnRK1 ACTIVITY

The maximum phosphorylation rate catalyzed by SnRK1 was estimated based on data from Zhang et al. (2009). The authors describe seedling SnRK1 activity without the addition of inhibitors to be about 2.25 nmol phosphate incorportated in AMARA peptide min^-1^ mgProtein^-1^ (Zhang et al., 2009) which we estimated to result in 1.35 μmol h^-1^ gFW^-1^ assuming a protein content of about 10 mg gFW^-1^ (∼1% w/w). As reported by Nunes et al. (2013b) replacing AMARA peptides by SPS did not significantly affect kinetic parameters. Hence, we assumed that these data on AMARA peptide phosphorylation are representative for our modeling approach which focused the SPS phosphorylation. Applying data on inhibitory constants for T6P (*K*_i,T6P_ = 0.005 mM), G1P (*K*_i,G1P_ = 0.48 mM) and G6P (*K*_i,G6P_> 1 mM; Nunes et al., 2013b) as well as the finding that experimental data were fitted best by a partial non-competitive mixed-type inhibition for T6P (Zhang et al., 2009), we then fitted the affinity, i.e., the dissociation constant K_M,SnRK1_ of SnRK1 to SPS peptide using a downhill simplex method in multidimensions as it is implemented in the Matlab^®^-based Systems Biology Toolbox 2 (Schmidt and Jirstrand, 2006). Simultaneously and applying the same methodology, the ratio of rate constants for partial inhibition by T6P, *k*_partial_ was estimated according to the inhibition kinetics presented by Nunes et al.(2013b). Best solutions were obtained for *K*_M,SnrK1_ = 10^-7^ mM and *k*_partial_ = 0.29.

### ODE PROGRAMMING, PARAMETER ESTIMATION AND SENSITIVITY ANALYSIS

The metabolic network shown in **Figure 1** was mathematically represented in a system of ODEs. The ODEs described the time dependent change in metabolite and enzyme levels as a function of enzymatic interconversion. Model parameters were estimated to simulate changes in metabolite levels during a 3 h supply of starved *Arabidopsis* seedlings with externally supplied sucrose with a concentration of 15 mM (Yadav et al., 2014). Kinetic parameters were numerically estimated by a downhill simplex method in multidimensions which is implemented in the Matlab^®^-based Systems Biology Toolbox 2 (Schmidt and Jirstrand, 2006). Upper and lower bounds for kinetic parameters were defined according to literature data (Osuna et al., 2007; Nägele et al., 2010, 2012). For reactions of PGI, PGM, and UGPase no explicit enzyme parameters were available. Instead, the reaction catalyzed by cFBPase, which was defined as the input reaction of the system, was considered to be the rate limiting step (Strand et al., 2000), i.e., resulting rates of PGI-, PGM,- and UGPase-driven reactions had to be lower than/equal to the maximum enzyme activity of cFBPase which was analyzed by Osuna et al. (2007). The resulting ODE model is provided with parameters to simulate dynamics of carbohydrate metabolism induced by external sucrose supply (Supplementary Information 1: Model_dynamic.txt) as well as to simulate a metabolic steady-state after 3 h of sucrose supply (Supplementary Information 2: Model_steady-state.txt). The model can be simulated and analyzed within the Matlab^®^-based Systems Biology Toolbox 2 (Schmidt and Jirstrand, 2006).

Sensitivity analysis was performed using the “SBsensdatastat” and the “SBsensstat” function implemented in the Systems Biology Toolbox 2 (Schmidt and Jirstrand, 2006). During this sensitivity analysis, model parameters are perturbed with a defined intensity (here: 1% of the parameter value). Afterward, simulation results of the perturbed system are compared to the non-perturbed system. In this way it is possible to estimate the impact of small parameter perturbations to model states, i.e., metabolite and protein concentrations and reaction rates.

## AUTHOR CONTRIBUTIONS

Thomas Nägele and Wolfram Weckwerth conceived and designed the study. Thomas Nägele performed ODE programming, modeling, *in silico* experiments and sensitivity analysis. Thomas Nägele and Wolfram Weckwerth wrote the manuscript.

## Conflict of Interest Statement

The authors declare that the research was conducted in the absence of any commercial or financial relationships that could be construed as a potential conflict of interest.
