# Supplementary material for: Mathematical modeling reveals that metabolic feedback regulation of SnRK1 and hexokinase is sufficient to control sugar homeostasis from energy depletion to full recovery
Source: Front Plant Sci. 2014 Jul 28;5:365. doi: 10.3389/fpls.2014.00365 (PMC4112813; doi:10.3389/fpls.2014.00365)
Supplement: Supplementary file 1 [file Data_Sheet1.PDF]

An approach of mathematical modeling connects plant sugar metabolism to posttranslational regulation of energy homeostasis

Running title: Linking sugar metabolism to energy homeostasis

Thomas Nägele* and Wolfram Weckwerth

Department of Ecogenomics and Systems Biology, University of Vienna, Althanstr. 14, 1090 Vienna, Austria

***Correspondence:**

Thomas Nägele

Department of Ecogenomics and Systems Biology

University of Vienna

Althanstr. 14, 1090 Vienna, Austria

Tel.: +43-1-4277-76556

Email: Thomas.Naegele@univie.ac.at

Supplementary Information 1: Model_dynamic.txt

```
********** MODEL NAME
The SnRK1 Hxk Interaction in Arabidopsis

********** MODEL NOTES

********** MODEL STATES
d/dt(F6P) = v_in-vPGI-vSPS+vFrcK
d/dt(G6P) = vPGI-vPGM+vGlcK-vTPS
d/dt(G1P) = vPGM-vUGPase
d/dt(UDPG) = vUGPase-vSPS-vTPS+v_SuSy
d/dt(T6P) = vTPS-vTPP
d/dt(Tre) = vTPP-vTH
d/dt(Suc) = vSPS-vInv-vSucExp-v_SuSy+v_ext
d/dt(Glc) = 2*vTH+vInv-vGlcK
d/dt(Fru) = vInv-vFrcK+v_SuSy
d/dt(E_SPS_act) = v6_pp-v6_snrk
d/dt(E_SPS_inact) = v6_snrk-v6_pp

F6P(0) = 0.01750320962888367
G6P(0) = 0.072583905299552165
G1P(0) = 0.01249777132495347
UDPG(0) = 0.024979960350361513
T6P(0) = 1.9918120483052173e-05
Tre(0) = 1.9911228651980612e-05
Suc(0) = 0.19963539889840759
Glc(0) = 0.20045346004139716
Fru(0) = 0.19882654047191375
E_SPS_act(0) = 1.86435e-06
E_SPS_inact(0) = 6.46087e-06

********** MODEL PARAMETERS
k_in_1 = 0.91046
k_in_2 = 1.27284
k_in_3 = 1.4054
k_in_4 = 1.38695
vmax_ext = 11.6877
Km_ext = 8.64137
kPGI = 18.4604
kPGM = 4.04061
kUGPase = 37.3299
KmSPSa = 0.0163982
KmSPSb = 0.543849
vmaxTPS = 4.20342
KmTPSa = 10.1775
KmTPSb = 6.73593
kTPP = 0.483907
vmaxTH = 1.35677
KmTH = 0.00132804
vmaxInv = 7.84844
KmInv = 1.00246
KiInva = 0.081579
KiInvb = 1.93433
vmaxFrcK = 7.76245
KmFrcK = 0.0197756
```

```
KiFrcK = 0.000162033
vmaxGlcK = 6.28199
KmGlcK = 0.454395
KiGlcK = 0.000342891
kSucExp = 17.7091
KmSucExp = 6.8451
k_SuSy = 0.000196116
Km_SuSy = 7.16566
k6_pp = 1.99446e+07
Km_pp = 66.4612
%

vmax1_snrk = 1.3500000000000001
Km_snrk = 9.9999999999999995e-08
k_snrk = 0.215
Ki_snrk_G6P = 2
Ki_snrk_G1P = 0.47999999999999998
Ki_snrk_T6P = 0.0050000000000000001
kSPS = 2980000

********** MODEL VARIABLES
Suc_Ext = interpcsSB([0,0.0000001,0.5,1.25,3],[0,15,15,15,15],time)

k_in  =interpcsSB([0,0.5,1.25,3],[k_in_1,k_in_2,k_in_3,k_in_4],time)


********** MODEL REACTIONS
vmax2_snrk = k_snrk*vmax1_snrk
v6_snrk =
((vmax1_snrk*(E_SPS_act))*(1+(vmax2_snrk*T6P/Ki_snrk_T6P)))/(Km_snrk
+((E_SPS_act)*(1+(T6P/Ki_snrk_T6P)+(((T6P*G1P)/(Ki_snrk_T6P*Ki_snrk_
G1P))+((T6P*G6P))/(Ki_snrk_T6P*Ki_snrk_G6P))+(G1P/Ki_snrk_G1P)+(G6P/
Ki_snrk_G6P))));
v6_pp = (k6_pp*E_SPS_inact)/(Km_pp+E_SPS_inact)
v_ext = (vmax_ext*Suc_Ext)/(Km_ext+Suc_Ext)
v_in = k_in
vPGI = kPGI*F6P
vPGM = kPGM*G6P
vUGPase = kUGPase*G1P
vSPS = (kSPS*E_SPS_act)*(F6P/(F6P+KmSPSa))*(UDPG/(UDPG+KmSPSb))
vTPS = (vmaxTPS)*(G6P/(G6P+KmTPSa))*(UDPG/(UDPG+KmTPSb))
vTPP = kTPP*T6P
vTH = (vmaxTH*Tre)/(KmTH+Tre)
vInv = (vmaxInv*Suc)/((KmInv*(1+Fru/KiInva)+Suc*(1+Glc/KiInvb)))
vFrcK = (vmaxFrcK*Fru)/((KmFrcK+Fru)*(1+F6P/KiFrcK))
vGlcK = (vmaxGlcK*Glc)/((KmGlcK+Glc)*(1+G6P/KiGlcK))
vSucExp = (kSucExp*Suc)/(KmSucExp+Suc)
v_SuSy = k_SuSy*Suc

********** MODEL FUNCTIONS


********** MODEL EVENTS


********** MODEL MATLAB FUNCTIONS
```

Supplementary Information 2: Model_steadystate.txt

```
********** MODEL NAME
The SnRK1 Hxk Interaction in Arabidopsis

********** MODEL NOTES

********** MODEL STATES
d/dt(F6P) = v_in-vPGI-vSPS+vFrcK
d/dt(G6P) = vPGI-vPGM+vGlcK-vTPS
d/dt(G1P) = vPGM-vUGPase
d/dt(UDPG) = vUGPase-vSPS-vTPS+v_SuSy
d/dt(T6P) = vTPS-vTPP
d/dt(Tre) = vTPP-vTH
d/dt(Suc) = vSPS-vInv-vSucExp-v_SuSy+v_ext
d/dt(Glc) = 2*vTH+vInv-vGlcK
d/dt(Fru) = vInv-vFrcK+v_SuSy
d/dt(E_SPS_act) = v6_pp-v6_snrk
d/dt(E_SPS_inact) = v6_snrk-v6_pp

F6P(0) = 0.040000000000000001
G6P(0) = 0.17500017089279929
G1P(0) = 0.01750001708927993
UDPG(0) = 0.047500046844346028
T6P(0) = 0.00070000150937491281
Tre(0) = 4.926333959119131e-07
Suc(0) = 4.24999330771929
Glc(0) = 3.9000752479569485
Fru(0) = 2.8999930774290301
E_SPS_act(0) = 6.6795906266346403e-06
E_SPS_inact(0) = 1.7204093733653591e-06

********** MODEL PARAMETERS
k_in_4 = 1.3837200000000001
vmax_ext = 14.4612
Km_ext = 16.9313
kPGI = 17.296500000000002
kPGM = 4.0550899999999999
kUGPase = 40.550899999999999
KmSPSa = 0.030104100000000002
KmSPSb = 0.71278600000000003
vmaxTPS = 4.5550800000000002
KmTPSa = 8.9595400000000005
KmTPSb = 8.0758799999999997
kTPP = 0.72896399999999995
vmaxTH = 2.0285899999999999
KmTH = 0.0019579599999999999
vmaxInv = 2.0124599999999999
KmInv = 8.9851399999999995
KiInva = 0.056246200000000003
KiInvb = 0.88563000000000003
vmaxFrcK = 6.8365400000000003
KmFrcK = 0.0106699
KiFrcK = 0.00010431500000000001
vmaxGlcK = 6.4566800000000004
```

```
KmGlcK = 0.72560000000000002
KiGlcK = 0.00058999400000000004
kSucExp = 27.684100000000001
KmSucExp = 11.4688
k_SuSy = 0.000104968
Km_SuSy = 7.0068400000000004
k6_pp = 33719200
Km_pp = 53.527500000000003
%
vmax1_snrk = 1.3500000000000001
Km_snrk = 9.9999999999999995e-08
k_snrk = 0.215
Ki_snrk_G6P = 2
Ki_snrk_G1P = 0.47999999999999998
Ki_snrk_T6P = 0.0050000000000000001
kSPS = 2980000
Suc_Ext = 15

********** MODEL VARIABLES


********** MODEL REACTIONS
vmax2_snrk = k_snrk*vmax1_snrk
v6_snrk =
((vmax1_snrk*(E_SPS_act))*(1+(vmax2_snrk*T6P/Ki_snrk_T6P)))/(Km_snrk
+((E_SPS_act)*(1+(T6P/Ki_snrk_T6P)+(((T6P*G1P)/(Ki_snrk_T6P*Ki_snrk_
G1P))+((T6P*G6P))/(Ki_snrk_T6P*Ki_snrk_G6P))+(G1P/Ki_snrk_G1P)+(G6P/
Ki_snrk_G6P))));
v6_pp = (k6_pp*E_SPS_inact)/(Km_pp+E_SPS_inact)
v_ext = (vmax_ext*Suc_Ext)/(Km_ext+Suc_Ext)
v_in = k_in_4
vPGI = kPGI*F6P
vPGM = kPGM*G6P
vUGPase = kUGPase*G1P
vSPS = (kSPS*E_SPS_act)*(F6P/(F6P+KmSPSa))*(UDPG/(UDPG+KmSPSb))
vTPS = (vmaxTPS)*(G6P/(G6P+KmTPSa))*(UDPG/(UDPG+KmTPSb))
vTPP = kTPP*T6P
vTH = (vmaxTH*Tre)/(KmTH+Tre)
vInv = (vmaxInv*Suc)/((KmInv*(1+Fru/KiInva)+Suc*(1+Glc/KiInvb)))
vFrcK = (vmaxFrcK*Fru)/((KmFrcK+Fru)*(1+F6P/KiFrcK))
vGlcK = (vmaxGlcK*Glc)/((KmGlcK+Glc)*(1+G6P/KiGlcK))
vSucExp = (kSucExp*Suc)/(KmSucExp+Suc)
v_SuSy = k_SuSy*Suc

********** MODEL FUNCTIONS


********** MODEL EVENTS


********** MODEL MATLAB FUNCTIONS
```
